# *De novo* filament formation by human hair keratins K85 and K35 follows a filament development pattern distinct from cytokeratin filament networks

**DOI:** 10.1002/2211-5463.13126

**Published:** 2021-04-03

**Authors:** Masaki Yamamoto, Yasuko Sakamoto, Yuko Honda, Kenzo Koike, Hideaki Nakamura, Toshihiko Matsumoto, Shoji Ando

**Affiliations:** ^1^ Faculty of Biotechnology and Life Science Sojo University Kumamoto Japan; ^2^ Faculty of Medicine Saga University Japan; ^3^ Hair Care Research Center KAO Corporation Tokyo Japan; ^4^ Faculty of Pharmaceutical Science Sojo University Kumamoto Japan

**Keywords:** ectodermal dysplasia, fluorescent protein, hair keratin, intermediate filament, macrofibril

## Abstract

In human hair follicles, the hair‐forming cells express 16 hair keratin genes depending on the differentiation stages. K85 and K35 are the first hair keratins expressed in cortical cells at the early stage of the differentiation. Two types of mutations in the gene encoding K85 are associated with ectodermal dysplasia of hair and nail type. Here, we transfected cultured SW‐13 cells with human K85 and K35 genes and characterized filament formation. The K85–K35 pair formed short filaments in the cytoplasm, which gradually elongated and became thicker and entangled around the nucleus, indicating that K85–K35 promotes lateral association of short intermediate filaments (IFs) into bundles but cannot form IF networks in the cytoplasm. Of the K85 mutations related to ectodermal dysplasia of hair and nail type, a two‐nucleotide (C^1448^T^1449^) deletion (delCT) in the protein tail domain of K85 interfered with the K85–K35 filament formation and gave only aggregates, whereas a missense mutation (233A>G) that replaces Arg^78^ with His (R78H) in the head domain of K85 did not interfere with the filament formation. Transfection of cultured MCF‐7 cells with all the hair keratin gene combinations, K85–K35, K85(R78H)–K35 and K85(delCT)–K35, as well as the individual hair keratin genes, formed well‐developed cytoplasmic IF networks, probably by incorporating into the endogenous cytokeratin IF networks. Thus, the unique *de novo* assembly properties of the K85–K35 pair might play a key role in the early stage of hair formation.

## Graphical abstract

We transfected SW‐13 cells with human hair keratin K85 and K35 genes. This pair formed short filaments in the cytoplasm, which gradually elongated, became thicker and entangled around the nucleus, indicating that K85–K35 promotes lateral association of short intermediate filaments into bundles but cannot form filament networks in the cytoplasm. Two K85 mutants related to ectodermal dysplasia were also tested.

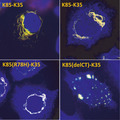

**Abbreviations**
AcGFP: *Aequorea coerulescens* GFP
DAPI: 4′, 6‐diamidino‐2‐phenylindole
DsRed: *Discosoma* sp. red fluorescent protein
IF: intermediate filament
KAP: keratin‐associated protein
MF: macrofibril
proto‐MF: proto‐macrofibril
RFP: red fluorescent protein
TEM: transmission electron microscopy
ULF: unit‐length filament

Keratins are the most complex subgroup of intermediate filament (IF) proteins [1, 2, 3]. Human IF proteins are encoded by at least 37 cytokeratin (epithelial keratin) genes, which are expressed in different types of epithelia. At least 16 hair keratin genes are expressed in hair‐ or nail‐forming cells, and one hair keratin gene is expressed in the filiform papillae of the tongue [3, 4, 5, 6, 7, 8, 9, 10, 11]. These keratins are subdivided into two types: type I keratins are relatively acidic, and type II keratins are neutral to basic. A type I and a type II keratin are required to form IFs with a width of 7–11 nm [12, 13].

The expression patterns of hair keratins and cytokeratins in human hair follicles have been determined [4, 5, 6, 7, 8, 19]. The tissue units outside the hair‐forming compartments are the outer root sheath, companion layer and inner root sheath, each of which expresses specific sets of type I and type II cytokeratins. By contrast, the hair‐forming compartments, such as the matrix (just above the germinative cells), cortex (differentiated from the matrix and constituting the bulk of hair fibers) and cuticle (surrounding the cortex) show complex expression patterns of hair keratins depending on hair differentiation. In the early stage of differentiation, the matrix/precortex begins to express hair keratin 35 [20] (abbreviated here as K35; the numbers 31–40 represent type I hair keratins [21, 22]) and hair keratin 85 [23] (K85; the numbers 81–86 represent type II hair keratins [21, 22]), while the early cuticle expresses K32, K35 and K85. During the middle stage of differentiation, the lower cortex begins to express K31, and the cuticle begins to express K82. Although the expression of K35 ceases somewhere near the top of the hair bulb, K31 expression continues until the upper cortex. The lower cortex also weakly expresses K38 in a randomly scattered pattern. At an advanced stage of differentiation, the middle to upper cortex shows almost simultaneous expression of K33a, K33b, K36, K81, K83 and K86, and finally, K34 and K39. The cortex of vellus hairs expresses K37 as well. The cuticle layer expresses K39 and K40. Such a complex pattern of hair keratin expression makes it difficult to precisely determine the specific type I and II hair keratin pairs used to form IFs, except for the first pair, K85–K35, expressed in the matrix/precortex.

In the cortex of a hair or wool fiber, hair keratin IFs are arrayed into bundles, in which each IF is surrounded by a matrix material composed of keratin‐associated proteins (KAPs) [3, 4, 24, 25, 26]. The supramolecular structures organized by hair keratin IF bundles and KAPs, termed macrofibrils (MFs), have a diameter of 200–500 nm and a maximum length of ~10 µm and fill the cortex by lateral and longitudinal assembly along the fiber [4, 37]. Transmission electron microscopy (TEM) studies of MF formation have revealed the appearance of hair keratin IF bundles in the lower cortex, i.e., proto‐macrofibril (proto‐MFs) with a width of 50–100 nm and a length of several hundred nanometers, some of which are attached to a desmosome, while others are isolated in the cytoplasm [4, 26, 28, 30, 31, 34, 35, 37]. Some TEM images of early proto‐MFs showed that distinctly separated IFs are arranged on a hexagonal basal lattice like a columnar hexagonal liquid crystal, with no matrix proteins interpolated between the filaments [4, 34, 35, 37], indicating that the early proto‐MFs composed of just a few type I and type II hair keratins are substantially complete before the numerous KAPs are expressed. In the middle to upper cortex, small proto‐MFs coalesce with each other into mature MFs [4, 26, 28, 30, 31, 34, 35]. The initiation of proto‐MFs appeared to be limited to the lower cortex, and the increases in keratin content in the middle to upper cortex result from increases in the size of existing proto‐MFs, rather than initiation of new ones [31]. Among the human KAPs, KAP8.1 and KAP11.1, which are rich in glycine–tyrosine and cysteine, respectively, are expressed first in the lower cortex [24]. Other KAPs, including KAP1–4 and KAP9, which are high or ultrahigh in sulfur content, are expressed primarily in the middle to upper cortex during the advanced stage of differentiation [24]. The KAPs likely infiltrate into the proto‐MFs and MFs, and oxidation of the cysteine residues in both the hair keratins and the KAPs takes place, giving rise to an extensive network of disulfide linkages [24].

Gene mutations that cause inherited hair diseases, such as monilethrix and ectodermal dysplasia of hair and nail type, have been identified in type II hair keratins [5]. Monilethrix is an autosomal dominant hair disease whose hallmark is a beaded appearance. Causative mutations occur mainly in K86 and occasionally in K81 and K83 [38, 39, 40]. Ectodermal dysplasia of hair and nail type is a congenital disorder characterized by hypotrichosis and nail dystrophy. In consanguineous families with autosomal recessive ectodermal dysplasia of hair and nail type, two homozygous mutations have been identified in K85 [41, 42]. One mutation is a G‐to‐A substitution that converts Arg^78^ to His (R78H) in the head domain of K85 [41, 42]. The other mutation deletes C and T nucleotides (delCT) from the Pro^483^ codon in the tail domain, resulting in a frameshift and a premature termination codon 18 amino acid residues downstream of the mutation [42]. The former mutation results in more severe phenotypes (complete alopecia and severe nail dystrophy) than the latter mutation [42]. The mechanisms by which these mutations in type II hair keratin genes lead to hair diseases remain unknown.

Although IF formation *in vivo* and *in vitro* has been well documented for cytokeratins [12, 13, 43], few studies on IF assembly of hair keratins have been undertaken. Yu *et al*. [44] reported that mouse type I hair keratin, Ha1 (current designation K31), and type II, Hb4 (K84), when transiently expressed individually in HeLa cells, readily incorporated into the endogenous cytokeratin IF networks. Manabe *et al*. [45] reported that Ha1 and Hb4, when transiently expressed individually or in combination in epithelial PtK2 cells, formed aggregates of ringlike structures around the nucleus, while in rat epidermal keratinocytes, Ha1 and Hb4 were able to colocalize with the endogenous cytokeratin IF networks. For *in vitro* IF assembly, Wang *et al*. [46] reported efficient IF assembly of a mouse hair keratin pair. Hofmann *et al*. [47] reported that *in vitro* IF formation of human hair keratins, K31, K83 and K86, differs from that of human cytokeratins because of the requirement of a relatively high ionic strength in the assembly reaction mixture. We have previously reported that affinities of *in vitro* heterotypic subunit interactions depend on the combination of type I (K35, K36 and K38) and type II (K81 and K85) keratins [48]. Furthermore, although K81 produced IFs by co‐polymerization with the type I hair keratins, the typical combination of K85 and K35 gave tight bundles of short IFs, which then further polymerized into large paracrystalline‐like assemblies [48]. These results indicate that K85 promotes lateral association rather than longitudinal elongation of short IFs. This was consistent with the hypothesis that the unique assembly property of K85 is important for formation of proto‐MFs in the early stage of differentiation of hair‐forming cells. Actually, K85 is the only type II hair keratin protein expressed at that differentiation stage [4, 5, 6, 8, 18, 19] and is likely to be responsible for formation of the proto‐MFs with no matrix proteins. However, there are no experimental data concerning the function of K85 in cells.

In this study, we transiently expressed human K85 [23] and K35 [49], which were fused with fluorescent proteins, in human MCF‐7 [50, 51] and SW‐13 cells [52, 53]. We then characterized their IF assembly properties using fluorescence microscopy. We observed that the hair keratin pair forms short filaments on their own in the cytoplasm of SW‐13 cells, which gradually elongate and then entangle around the nucleus, but never produces long IF networks that spread throughout the cytoplasmic space, as usually observed for other IF proteins. Two K85 mutants related to ectodermal dysplasia of hair and nail type were also expressed in combination with K35 in cultured cells to assess the effects of the mutations on K85 function. We observed that at least one of the K85 mutants lacked filament formation competence. These results indicate a unique IF assembly function of the K85–K35 pair that is intrinsically different from the functions of cytokeratins.

## Materials and methods

### Plasmid construction

DNAs encoding human K35 (NM_002280, 1.4 kb) [49], K85 (NM_002283, 1.5 kb) [23] and the two K85 mutants related to ectodermal dysplasia of hair and nail type [41, 42] were chemically synthesized by GenScript (Piscataway, NJ, USA) and Fasmac (Atsugi, Japan). In the K85(R78H) mutant gene, the codon for Arg^78^ (CA ^233^C, numbering from the initiation codon) was replaced with the codon for His (CG ^233^C). In the K85(delCT) gene, two nucleotides (CT) in the Pro^483^ codon (CC ^1448^ T ^1449^) were deleted. The *Hind*III site (AAG ^114^CTT) and the *Bam*HI site (GG ^615^ATCC) in the K35 gene were silently mutated to AAA ^114^CTT and GA ^615^ATCC, respectively. To subclone the synthetic hair keratin genes into the expression vectors, pAcGFP1–Hyg‐N1 and pDsRed–Monomer‐N1 (Takara Bio USA, Mountain View, CA, USA), we added an extra DNA sequence (5′‐CTCGAGCTCAAGCTTGCCACC‐3′) containing an *Xho*I site and a *Hind*III site (both underlined) followed by the Kozak sequence to the 5′ of the initiation codons of the K85 and K35 genes. The termination codons of the hair keratin genes were replaced with GGG encoding Gly and followed by 5′‐TCGACGGTACCGCGGGCCCGGGATCCACCGGTC‐3′ containing a *Bam*HI site (underlined) and encoding a linker sequence (Ser‐Thr‐Val‐Pro‐Arg‐Ala‐Arg‐Asp‐Pro‐Pro‐Val) that connects the hair keratin genes in‐frame to the fluorescent protein genes in the expression vectors. The engineered hair keratin genes were cloned using appropriate vectors and confirmed by sequencing. After digestion of the constructs with *Xho*I and *Bam*HI, the resulting K85 genes and the K35 gene were subcloned into the pAcGFP1–Hyg‐N1 and pDsRed–Monomer‐N1 vectors, respectively. The constructs, pAcGFP1–Hyg‐N1–K85 and pDsRed–Monomer‐N1–K35, were purified using an EndoFree Plasmid Maxi Kit (Qiagen, Hilden, Germany) and then confirmed by sequencing.

The K85(△Head) and K85(△tail) genes, in which the head domain‐ and tail domain‐coding sequences were deleted, respectively, were prepared by in‐fusion cloning [54]. For deletion of the head domain‐coding sequence, inverse PCR was carried out using pAcGFP1–Hyg‐N1–K85 as a template, a forward primer (5′‐CTCAAGCTTGCCACCATGGAGAAGGAGCAGATCAAGTCCCTCAA‐3′) containing the vector‐specific 15‐base extension sequence (underlined) followed by the initiation codon and the rod domain‐coding sequence of K85, and a reverse primer (5′‐GGTGGCAAGCTTGAGCTCGAGATCTGAGT‐3′) containing the vector‐specific complementary sequence. To delete the tail domain‐coding sequence, we performed inverse PCR using pAcGFP1–Hyg‐N1–K85 as a template, a forward primer (5′‐ GAGGAACACAGGCTGGGGTCGACGGTACCGCGGGCCCGGGAT‐3′) containing a 15‐base extension sequence corresponding to the C‐terminal region of the rod domain of K85 (underlined) and the vector sequence for the linker region between K85 and the fluorescent protein, and a reverse primer (5′‐CAGCCTGTGTTCCTCGCCCTCCAGCAGGCGCCTGT‐3′) containing the complementary sequence for the C‐terminal region of the rod domain. The inverse PCR products were purified by agarose gel electrophoresis followed by gel extraction. The samples were treated with In‐Fusion HD Enzyme Premix (Takara Bio USA) for 15 min at 50℃, placed on ice and then transformed into *Escherichia coli* DH5α competent cells (Toyobo, Osaka, Japan). The resultant constructs, pAcGFP1–Hyg‐N1–K85(△Head) and pAcGFP1–Hyg‐N1–K85(△Tail), were purified using the plasmid purification kit and confirmed by sequencing.

The cDNAs of mouse type I cytokeratin 18 (K18, acc. BC089022) [55, 56] and type II cytokeratin 8 (K8, acc. M21836) [57, 58] were amplified by PCR using an NIH3T3 cell cDNA library and inserted into pDsRed–Monomer‐N1 and pAcGFP1–Hyg‐N1, respectively, using the *Xho*I and *Bam*HI sites as described for K35 and K85. The constructs pDsRed–Monomer‐N1–K18 and pAcGFP1–Hyg‐N1–K8 were purified as described earlier and confirmed by sequencing.

### Cell culture and transfection

MCF‐7 cells (a human mammary carcinoma cell line) [50, 51] and SW‐13 cells (a human adenocarcinoma cell line derived from the adrenal cortex) [52, 53] were purchased from the Japanese Collection of Research Bioresources Cell Bank (Ibaraki City, Japan). The cells were grown in Dulbecco's modified Eagle’s medium/Ham’s F‐12 medium (Fujifilm Wako Pure Chemical, Osaka, Japan) supplemented with 10% fetal calf serum in a 5% CO_2_ atmosphere at 37 °C. SW‐13 cells expressing only vimentin as an endogenous IF protein were cloned [53], confirmed by SDS/PAGE and western blotting, and used for subsequent experiments. Transfection of MCF‐7 and SW‐13 cells with plasmids was performed by lipofection [59] using ScreenFect A Plus (Fujifilm Wako Pure Chemical), according to the manufacturer's protocol.

### Immunocytochemistry

Cells grown on glass coverslips were fixed in 4% formaldehyde in PBS at room temperature for 15 min and permeabilized with 0.1% Triton X‐100 in PBS at room temperature for 5 min. To detect endogenous vimentin in SW‐13 cells, we incubated the cells overnight at 4 °C with rabbit anti‐vimentin antibody (1 : 100 dilution) (Cell Signaling Technology, Danvers, MA, USA). To detect endogenous cytokeratin 8 in MCF‐7 cells, we incubated the cells overnight at 4 °C with rabbit anti‐(cytokeratin 8) antibody (1 : 200 dilution) (Abcam, Cambridge, UK). After washing three times with PBS, the cells were incubated for 1 h with Alexa Fluor 405‐conjugated goat anti‐rabbit IgG antibody (1 : 200 dilution) (Abcam). When required, cell nuclei were stained with 1 µg·mL^−1^ 4', 6‐diamidino‐2‐phenylindole (DAPI) (Dojindo, Kumamoto, Japan) for 5 min at room temperature. Fluorescently labeled cells were examined using an Olympus BX53 fluorescence microscope (Olympus, Tokyo, Japan) and a Nikon C1 confocal microscope (Nikon, Tokyo, Japan).

### Western blotting

Cells grown in a dish were treated with 10% trichloroacetic acid on ice for 15 min, scraped off the dish bottom and collected by centrifugation. The collected cells were disrupted in SDS sample buffer containing 5 mm DTT with brief sonication. Cell lysate aliquots were loaded on 7.5% polyacrylamide gels and resolved by SDS/PAGE. The proteins were transferred onto polyvinylidene difluoride membranes (Thermo Fisher Scientific, Waltham, MA, USA) and detected by immunochemical staining. Guinea pig anti‐K85 serum (Progen Biotechnik, Heidelberg, Germany), guinea pig anti‐K35 serum (Progen Biotechnik), rabbit anti‐vimentin IgG (Cell Signaling Technology), rabbit anti‐cytokeratin 8 IgG (Abcam), rabbit anti‐GFP IgG (MBL, Nagoya, Japan) and rabbit anti‐RFP IgG (MBL) were used as primary antibodies. Anti‐GFP IgG and the anti‐RFP IgG can detect the *Aequorea coerulescens* GFP (AcGFP1) and the *Discosoma* sp. red fluorescent protein (DsRed), respectively. Alkaline phosphatase‐conjugated goat anti‐(guinea pig IgG) (Abcam) and alkaline phosphatase‐conjugated goat anti‐rabbit IgG (Abcam) were used as secondary antibodies. The immunoreactive bands were visualized using CDP‐star (Sigma‐Aldrich, St. Louis, MO, USA), an alkaline phosphatase substrate.

## Results

**Fig. 1:**
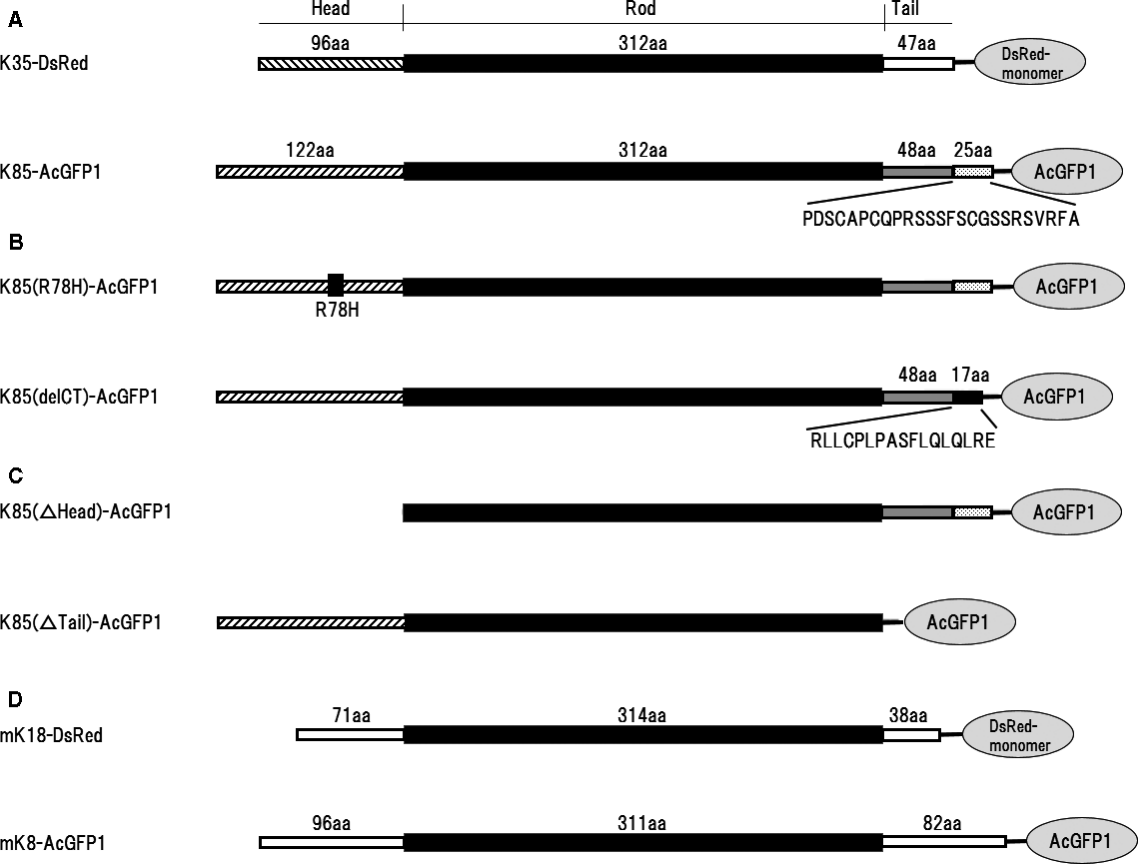
Schematic representation of the tripartite domain structures of human hair keratins expressed in cultured cells. (A) Type I hair keratin K35 and type II hair keratin K85 were C‐terminally tagged with DsRed‐monomer and AcGFP1 fluorescent proteins, respectively. The central rod domain (312 amino acids) is flanked by the N‐terminal head domain (96 amino acids for K35 and 122 for K85) and the C‐terminal tail domain (47 for K35 and 73 for wild‐type K85). (B) The K85 mutant protein, K85(R78H), of ectodermal dysplasia of hair and nail type contains histidine in place of arginine (filled box) at the 78th position from the N‐terminal methionine. The other mutant protein, K85(delCT), of ectodermal dysplasia of hair and nail type contains a 17‐amino acid sequence in the C‐terminal region of the tail domain in place of the 25‐amino acid sequence of the wild‐type, the sequences of which are shown beneath the schematic drawing for comparison. (C) K85(△Head) and K85(△Tail) lack all amino acid residues of the head and tail domains, respectively. (D) As a control, mouse type I cytokeratin K18 and type II cytokeratin K8 were also tagged with DsRed‐monomer and AcGFP1 fluorescent proteins, respectively.

To study filament assembly properties of the human hair keratin pair, K85 and K35, they were transiently expressed as fusion proteins with GFPs (AcGFP1) and RFPs (DsRed‐monomer), respectively (Fig. 1A), in cultured cells. Two K85 mutants for ectodermal dysplasia of hair and nail type, abbreviated here as K85(R78H) and K85(delCT) (Fig. 1B) [41, 42], were also expressed as fusion proteins with GFP. To characterize the significance of the head and tail domains of K85 in filament formation, we also expressed two K85 mutants lacking either the head domain [K85(△Head)] or the tail domain [K85(△Tail)] as fusion proteins with GFP (Fig. 1C). As controls, mouse cytokeratins 8 and 18 were also expressed as fusion proteins with GFPs and RFPs, respectively (Fig. 1D).

For transient expression of the hair keratins, we selected SW‐13 and MCF‐7 cells. MCF‐7 cells are simple epithelial cells of human mammary carcinoma origin, and they endogenously express cytokeratins 8, 18 and 19 [50, 51]. SW‐13 cells are derived from a nonepithelial human adrenal cortex carcinoma and lack endogenous IF proteins [52, 53]. Nevertheless, a few SW‐13 cells have been reported to express vimentin, a type III IF protein [53], which forms a homodimer and then polymerizes into IF networks that are distinct from cytokeratin IF networks and does not co‐polymerize with cytokeratins *in vivo* or *in vitro* [12, 13, 60]. We cloned a vimentin‐expressing SW‐13 cell and then used the cloned cells because these cells offer the opportunity to test for hair keratin assembly on a *de novo* basis and provide endogenous vimentin IFs as a reference of cytoplasmic IF arrays.

**Fig. 2:**
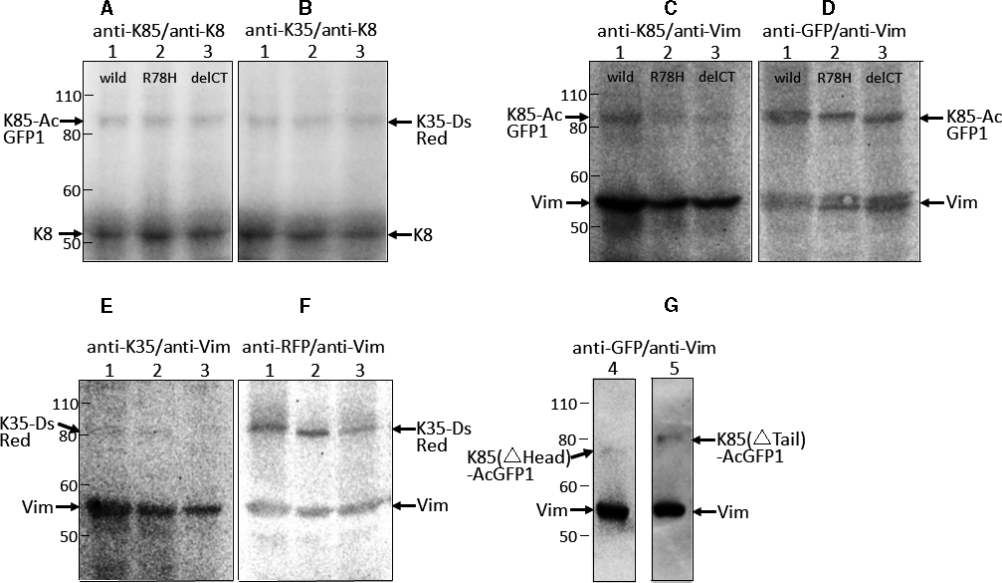
Western blot analysis of transiently expressed hair keratins K85 and K35 in cultured cells. (A–F) Pairs of K85–K35 (lane 1), K85(R78H)–K35 (lane 2) and K85(delCT)–K35 (lane 3) were expressed in MCF‐7 cells (A, B) or SW‐13 cells (C–F). (G) Pairs of K85(△Head)–K35 (lane 4) and K85(△tail)–K35 (lane 5) were expressed in SW‐13 cells. The cell lysates were resolved by SDS/PAGE on 7.5% polyacrylamide gels and transferred onto polyvinylidene difluoride membranes. (A, B) The K85 proteins, K35 and endogenous cytokeratin 8, were detected in the lysates of MCF‐7 cells using anti‐K85 serum, anti‐K35 serum and anti‐(cytokeratin 8) IgG as primary antibodies, respectively. (C, D) The K85 proteins in the lysates of SW‐13 cells were detected using anti‐K85 serum (C) or anti‐GFP IgG (D), while endogenous vimentin was detected using anti‐vimentin IgG. (E, F) K35 in the SW‐13 cell lysates was detected using anti‐K35 serum (E) or anti‐RFP IgG (F). (G) K85(△Head) (lane 4) and K85(△tail) (lane 5) were detected in the lysates of SW‐13 cells using anti‐GFP IgG. (E, F) K35 in the SW‐13 cell lysates was detected using anti‐K35 Ig (E) or anti‐RFP Ig (F). (G) K85(△Head) (lane 4) and K85(△tail) (lane 5) were detected in the lysates of SW‐13 cells using anti‐GFP Ig. The immunoreactive bands were visualized using alkaline phosphatase‐conjugated secondary antibodies and CDP‐star as a substrate. The arrows indicate the positions of wild‐type or mutant K85–AcGFP1, K35–DsRed, endogenous cytokeratin 8 and vimentin. Protein size (kDa) is indicated on the left in each figure. K8, cytokeratin 8; Vim, vimentin.

The hair keratin and cytokeratin genes were transfected into cultured cells by lipofection [59]. Western blot analysis of the transfected cells using a specific anti‐K85 antibody or an anti‐GFP antibody showed that K85 (theoretical value: 56 kDa), K85(R78H) (56 kDa), K85(delCT) (55 kDa), K85(△Head) (43 kDa) and K85(△Tail) (49 kDa) were expressed as fusion proteins with the AcGFP1 protein (27 kDa), with a total molecular mass of 83, 83, 82, 70 and 76 kDa, respectively (Fig. 2A,C,D,G). Similarly, the use of a specific anti‐K35 antibody or an anti‐RFP antibody showed that K35 (51 kDa) was expressed as a fusion protein with the DsRed‐monomer protein (26 kDa), with a total molecular mass of 77 kDa (Fig. 2B,E,F). Transfection of the K85 and K35 constructs at a ratio of 1 : 1 into SW‐13 cells and MCF‐7 cells resulted in the expression of K85 and K35 proteins at similar levels (Fig. 2A vs. Fig. 2B; Fig. 2C,D vs. Fig. 2E,F).

**Fig. 3:**
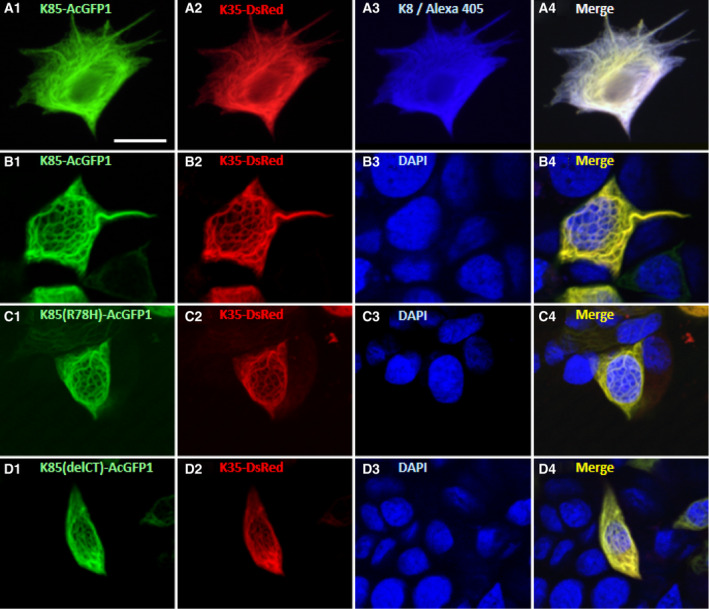
Expression of hair keratin K85 and K85 mutants in combination with K35 in MCF‐7 cells. The hair keratin pairs, K85–K35 (A, B), K85(R78H)–K35 (C) and K85(delCT)–K35 (D), were transiently expressed in MCF‐7 cells. Fluorescence images of the K85 proteins tagged with AcGFP1 [A‐1: K85–AcGFP1, B‐1: K85–AcGFP1, C‐1: K85(R78H)–AcGFP1, D‐1: K85(delCT)–AcGFP1], K35 tagged with DsRed (A‐2, B‐2, C‐2, D‐2), endogenous cytokeratin 8 immunologically visualized with Alexa Fluor 405 (A‐3) and nuclei stained with DAPI (B‐3, C‐3, D‐3) were observed and merged (A‐4, B‐4, C‐4, D‐4). Note that all three hair keratin pairs, K85–K35 (A, B), K85(R78H)–K35 (C) and K85(delCT)–K35 (D), formed IF networks spreading throughout the cytoplasmic space. Note that K85 (A‐1) and K35 (A‐2) appeared to colocalize with the endogenous cytokeratin 8 IF networks (A‐3), as shown in the merged image (A‐4). Scale bars: 20 µm.

We characterized filament formation of the exogenously expressed hair keratin proteins using fluorescence microscopy. As a control experiment, cells transfected with AcGFP1 or DsRed proteins resulted in unstructured fluorescence throughout cells (data not shown). In contrast, expression of the hair keratin pairs K85–K35, K85(R78H)–K35 or K85(delCT)–K35 in MCF‐7 cells resulted in the formation of IF networks spreading throughout the cytoplasmic space, as shown in Fig. 3. Indirect immunofluorescence staining of endogenous cytokeratin K8 and merging this image with that of K85–K35 indicated that K85 and K35 colocalized with the endogenous cytokeratin IF networks (Fig. 3A). Expression of individual hair keratin proteins, K85, K85(R78H), K85(delCT) or K35 also resulted in similar cytoplasmic filament formation (data not shown). These results indicate that the exogenously expressed K85–K35 pair and the individual hair keratin proteins integrate into preexisting cytokeratin IF networks and do not disturb them, as observed for exogenously expressed mouse hair keratins in cultured cells harboring endogenous cytokeratins [44, 45].

**Fig. 4:**
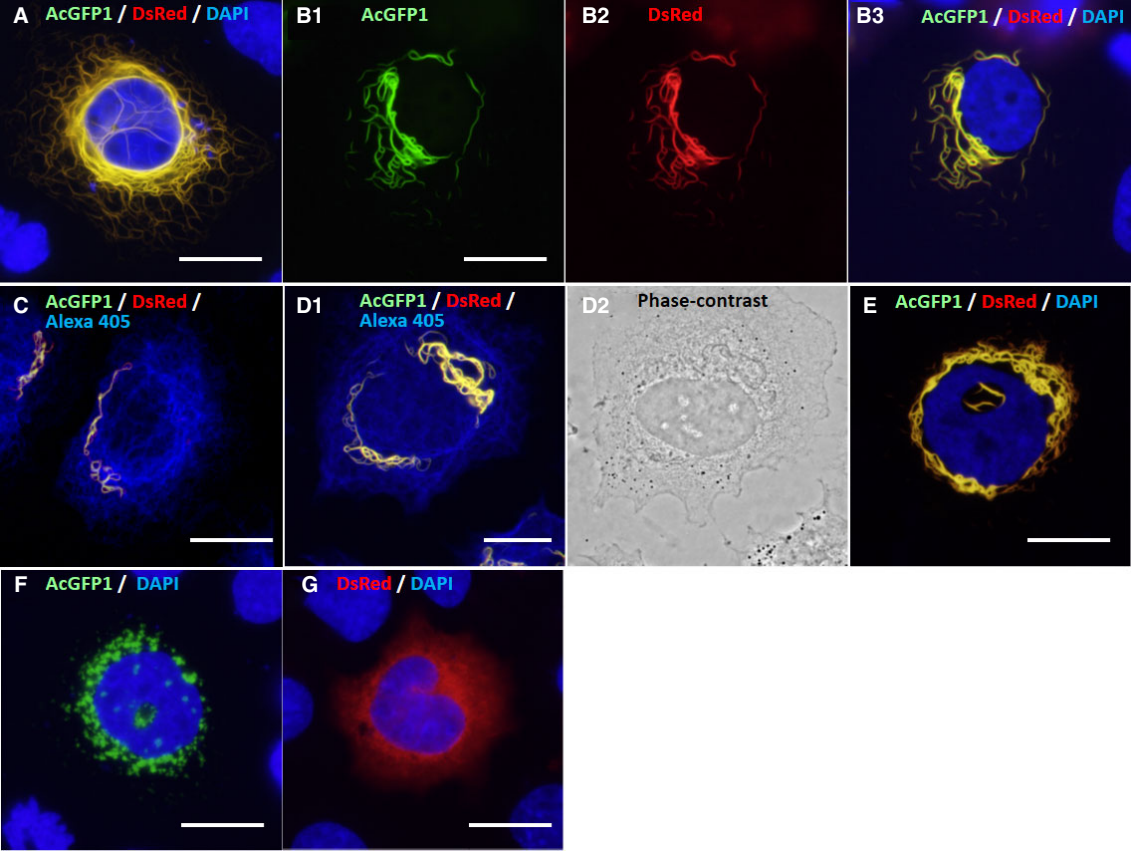
Expression of hair keratins K85 and K35 in SW‐13 cells. (A) As a control, mouse cytokeratin K8 tagged with AcGFP1 (K8–AcGFP1) and K18 tagged with DsRed (K18–DsRed) were exogenously coexpressed in SW‐13 cells. After cell nuclei were stained with DAPI (blue), the fluorescence images were merged. Note that the K8–K18 pair formed well‐developed IF networks (yellow) spreading throughout the cytoplasmic space. (B) Exogenously coexpressed hair keratins, K85–AcGFP1 (B‐1, green) and K35–DsRed (B‐2, red), co‐polymerized and formed relatively short filaments (yellow) in the cytoplasm and around the nucleus (blue) (B‐3). (C) Confocal microscopy fluorescence images of coexpressed K85–AcGFP1 and K35–DsRed were merged with endogenous vimentin IFs visualized with Alexa Fluor 405. The K85–K35 pair formed short filaments (yellow), while the endogenous vimentin IFs were spread throughout the cytoplasmic space (blue). Localization of the hair keratin filaments was distinct from the endogenous vimentin IFs. (D‐1) Another fluorescence microscopy image of K85–K35 pair filaments (yellow) and endogenous vimentin IFs (blue). Note that the filaments of the K85–K35 pair elongated, became thicker and got entangled. (D‐2) Phase‐contrast image of (D‐1). (E) Accumulated K85–K35 pair filaments (yellow) became entangled around the nucleus (blue). (F) Sole expression of K85–AcGFP1 produced aggregates (green) around the nucleus (blue). (G) Sole expression of K35–DsRed produced fine aggregates (red) in the cytoplasmic space. Scale bars: 20 µm.

In SW‐13 cells, when the mouse cytokeratin pair K8–K18 was expressed exogenously as a control, it formed well‐developed IF networks throughout the cytoplasmic space (Fig. 4A) and as previously reported [61, 62]). In contrast, the K85–K35 pair formed relatively short filaments in the cytoplasm, especially around the nucleus (Fig. 4B,C), which was apparently different from the IF networks of the K8–K18 pair. In contrast, endogenous vimentin formed authentic IF networks spreading throughout the cytoplasmic space (Fig. 4C,D). Further characterization revealed that most of the short filaments observed in the cytoplasmic space were initially 2–3 µm in length (Fig. 4B), then gradually elongated and became thicker, and then got entangled around the nucleus (Fig. 4C–E). For the duration of our observations, the K85–K35 pair never formed IF networks that spread throughout the cytoplasmic space. Thus, the K85–K35 pair exogenously expressed in SW‐13 cells showed a unique filament development pattern that is intrinsically different from those of authentic IFs, such as cytokeratins and vimentin. When K85 and K35 were expressed individually in SW‐13 cells, they produced no filamentous structures (Fig. 4F,G). K85 gave globular aggregates of various sizes around the nucleus (Fig. 4F), whereas K35 formed fine aggregates compared with those of K85 (Fig. 4G).

**Fig. 5:**
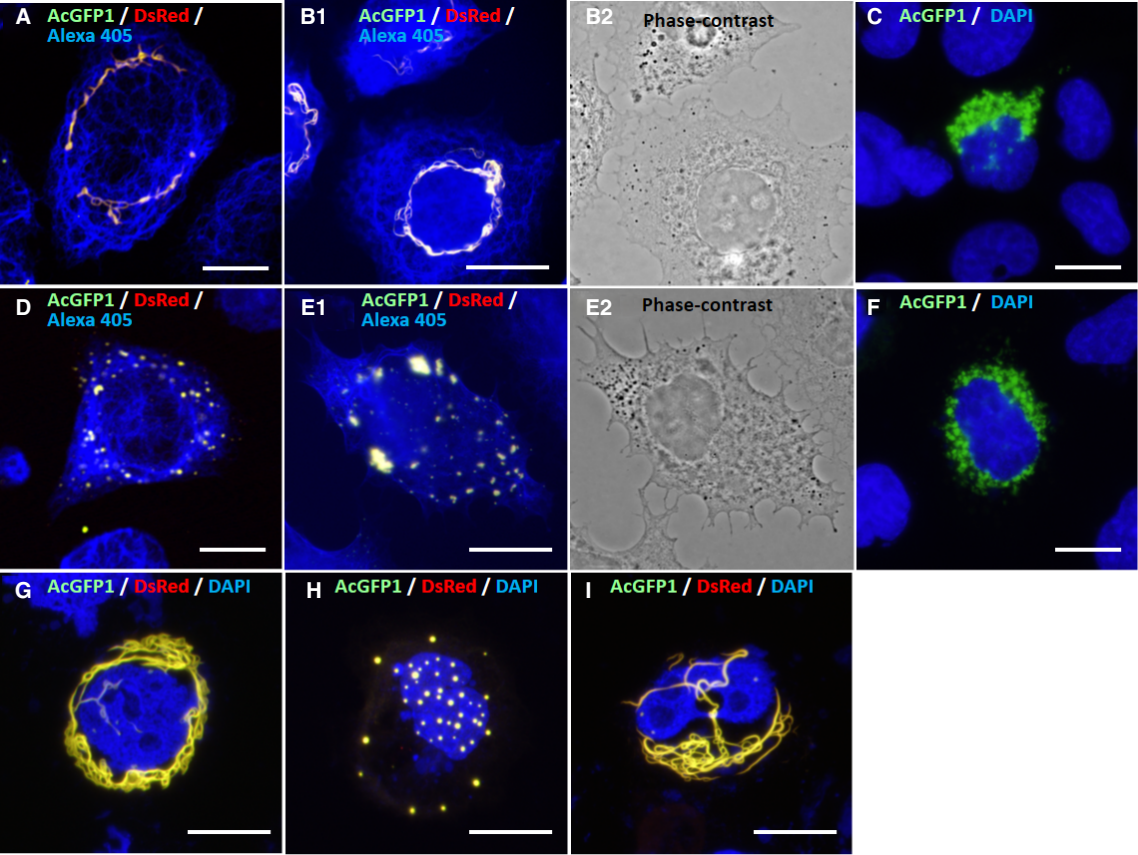
Expression of hair keratin K85 mutants and K35 in SW‐13 cells. (A) Confocal microscopy fluorescence images of exogenously coexpressed K85(R78H)–AcGFP1 and K35–DsRed were merged with endogenous vimentin IFs (blue) visualized immunologically with Alexa Fluor 405. This pair formed filaments (yellow) similar to those of wild‐type K85–K35 (Fig. 4B–E). (B‐1) Fluorescence microscopy images of the K85(R78H)–AcGFP1, K35–DsRed and endogenous vimentin IFs visualized with Alexa Fluor 405 were merged. Note that the R78H mutation in K85 did not interfere with filament formation. (B‐2) Phase‐contrast image of (B‐1). (C) Sole expression of K85(R78H)–AcGFP1 produced aggregates (green) around the nucleus (blue, DAPI). (D) Confocal microscopy images of exogenously coexpressed K85(delCT)–AcGFP1 and K35–DsRed and endogenous vimentin IFs visualized with Alexa Fluor 405 (blue) were merged. Note that K85(delCT)–AcGFP1 and K35–DsRed co‐polymerized but gave only aggregates (yellow). (E‐1) Fluorescence microscopy images of K85(delCT)–AcGFP1, K35–DsRed and the endogenous vimentin IFs (blue, Alexa Fluor 405) were merged. Note that the delCT mutation in K85 interfered with filament formation and gave only aggregates (yellow). (E‐2) Phase‐contrast image of (E‐1). (F) Sole expression of K85(delCT)–AcGFP1 produced only aggregates (green) around the nucleus (blue, DAPI). (G) Fluorescence microscopy images of exogenously co‐expressed K85(delCT)–AcGFP1, wild‐type K85–AcGFP1 and K35–DsRed were merged. Note that the presence of wild‐type K85 restored filament formation (yellow), when compared with (D) or (E‐1). (H) Fluorescence microscopy images of exogenously coexpressed K85(△Head)–AcGFP1, K35–DsRed and DAPI‐stained nucleus (blue) were merged. Note that deletion of the head domain of K85 resulted in failure of filament formation and gave only aggregates (yellow). (I) Fluorescence microscopy images of exogenously coexpressed K85(△Tail)–AcGFP1 and K35–DsRed and the nucleus (blue) were merged. Deletion of the tail domain of K85 did not interfere with filament formation. Scale bars: 20 µm.

The K85(R78H)–K35 pair also formed short filaments that gradually elongated and became thicker around the nucleus in SW‐13 cells (Fig. 5A,B). However, the K85(delCT)–K35 pair failed to form filamentous structures and gave only dot‐like aggregates in the cytoplasm (Fig. 5D,E). Interestingly, however, when wild‐type K85 and K85(delCT) were expressed simultaneously in combination with K35 in SW‐13 cells, they produced similar filaments to those observed for the wild‐type K85–K35 pair (Fig. 5G). Thus, the presence of wild‐type K85 compensated for the incompetence of the K85(delCT)–K35 pair in filament formation. Again, when K85(R78H) and K85(delCT) were expressed individually in SW‐13 cells, they produced no filamentous structures (Fig. 5C,F).

To assess the significance of the head and tail domains of K85 in filament formation, K85(△Head) or K85(△Tail) was expressed in combination with K35 in SW‐13 cells (Fig. 5H,I). The K85(△Head)–K35 pair failed to form filaments and gave only aggregates in the cytoplasm (Fig. 5H). In contrast, the K85(△Tail)–K35 pair produced filaments similar to those of the wild‐type K85–K35 pair (Fig. 5I). These results indicate that the head domain of K85 is indispensable for filament formation, whereas the tail domain can be removed without an apparent loss of filament formation.

## Discussion

In this study, we observed that exogenously coexpressed human hair keratins, K85 and K35, form a heterodimer that, on its own, propagates to form short filaments in the cytoplasm of SW‐13 cells, while the individual proteins did not form any filament structures. The short filaments gradually elongated, became thicker and finally became entangled around the nucleus, but they never formed filamentous networks spreading throughout the cytoplasmic space. Of the two K85 mutants related to ectodermal dysplasia of hair and nail type, K85(delCT) was unable to form filaments, while K85(R78H) unexpectedly formed filaments by co‐polymerization with K35. In MCF‐7 cells, all the hair keratin pairs, K85–K35, K85(R78H)–K35 and K85(delCT)–K35, as well as the individual proteins, formed well‐developed cytoplasmic IF networks, probably by incorporating into the endogenous cytokeratin IF networks. Thus, functional characteristics in cells of the human hair keratin pair of K85 and K35 were revealed in this study.

Several *in vitro* and *in vivo* experiments have shown that IF proteins, including cytokeratins and vimentin, readily form short IFs, named ‘unit‐length’ filaments (ULFs), which have a width of ~16 nm and a length of ~60 nm and which undergo rapid longitudinal annealing to each other to form mature IFs with a width of 7–11 nm and a length of several micrometers [13, 43, 63, 64]. In contrast, our previous *in vitro* assembly experiments of hair keratins revealed that, among the examined combinations of recombinant human type I and type II hair keratins, the K85–K35 pair readily formed short IFs with a width of about 7 nm and lengths of 200–400 nm, which tended to associate laterally and concatenate longitudinally to form short IF bundles with widths of 40–100 nm and lengths of 300–400 nm [48]. They then further associated with each other to form large paracrystalline‐like assemblies [48]. Although the short, thick filaments of around 2–3 µm in length observed in SW‐13 cells were longer than the short IF bundles observed in our previous *in vitro* assembly experiments, the results obtained here support the view that the K85–K35 pair tends to form short IF bundles by promoting the lateral association of short IFs, rather than forming long IFs by longitudinal elongation of short IFs.

TEM analysis by others of early‐stage MF formation in the lower cortex of human, sheep and mouse hair follicles has revealed the appearance of short hair keratin IF bundles, i.e., proto‐MFs, with a width of 50–100 nm and a length of several hundred nanometers [4, 26, 28, 30, 31, 34, 35, 37]. Some of these bundles are located in the cytoplasmic space, and others are associated with desmosomes of a cortical cell [4, 26, 28, 30, 31, 34, 35, 37]. The gene expression patterns reported so far suggest that K85 is the single type II hair keratin responsible for early‐stage proto‐MF formation through heterotypic subunit interaction with the type I hair keratin, K35 and/or K31 [4, 5, 6, 7, 8, 11, 17, 18, 19]. Some high glycine–tyrosine KAPs and high sulfur KAPs also start to be expressed in cortical cells at the early stage of differentiation [24], although there is still some debate about whether the hair keratins are expressed before the KAPs [24, 65, 66]. Some TEM studies have shown that early‐stage proto‐MFs can occur without interposing of KAPs (as matrix proteins) between the short IFs and also that these proto‐MFs appear to coalesce with each other into larger filamentous structures [4, 34, 35, 37]. The results obtained here support the view that hair keratins, such as the K85–K35 pair, can form the early‐stage proto‐MFs without interposing of KAPs in cortical cells. Thus, the unique ability of filament development observed for the K85–K35 pair in transfected SW‐13 cells might be critical for early‐stage proto‐MF formation in differentiating cortical cells. It seems reasonable for hair keratins to form short IF bundles at the beginning of MF formation and then to connect them longitudinally and laterally to form mature MFs, rather than to bundle long IFs into MFs. In addition, the inability of the K85–K35 pair to form IF networks that spread throughout a cytoplasmic space might be desirable because IF networks would obstruct MF formation in cells.

As an explanation of proto‐MF formation in cortical cells, a mesophase (lyotropic liquid crystal) separation model has been proposed from detailed TEM data and thermodynamic theories [26, 34, 35]. In this model, the abundant hair keratin pair, K85 and K31, builds up a high concentration of ULFs in the lower cortex cells, and any further lengthening of the ULFs into short IFs will quickly result in the formation of a concentrated oriented phase, the mesophase, appearing initially as spindle‐shaped tactoids, i.e., early‐stage proto‐MFs. It is hypothesized that incorporation of K35 into the ULFs by subunit interchange with K31 is a trigger for the initiation of ULF lengthening because the large N‐terminal head domain (96 amino acids) of K35 compared with the small head domain (55 amino acids) of K31 causes thermodynamic conversion of the ULFs from a stable (blocky) form to a shaggy form that can concatenate with each other [26, 35]. Although K31 was not tested in this study, the results obtained here and our previous *in vitro* data [48] are consistent with the earlier model in that the K85–K35 pair readily forms the short IF bundles on their own and they can gradually elongate in cells.

Comparison of the amino acid composition of hair keratins, including K85, K35 and K31, with those of cytokeratins shows that although there are no striking differences in the central rod domains, there are distinct variations within both the N‐terminal head and C‐terminal tail domains: the terminal domains of hair keratins are abundant in prolines and cysteines, whereas those of epidermal and simple‐type epithelial keratins are enriched in glycine–phenylalanine and basic–acidic residues, respectively [67]. The terminal domains of hair keratins might play a substantial role in determining not only the structures of filaments but also their rigidity and intracellular localization through interactions with other cellular components [1, 2, 67, 68].

The two K85 gene mutations that cause ectodermal dysplasia of hair and nail type showed different effects on filament formation in SW‐13 cells. It was surprising that the delCT mutation in the tail domain of K85 resulted in the loss of filament formation in SW‐13 cells because tail domains of IF proteins, including cytokeratins, are largely dispensable for IF assembly [12, 13, 69, 70]. However, the K85(△Tail)–K35 pair produced filaments similar to those of the wild‐type K85–K35 pair in SW‐13 cells, suggesting that the tail domain of K85 is dispensable for filament formation. Hence it is difficult to clearly explain the disrupted filament formation of the K85(delCT) mutant. The delCT mutation in the C‐terminal sequence of the tail domain results in an increased number of leucine residues (6 of the 17 mutated residues are leucine) compared with the native sequence (Fig. 1). Secondary structure prediction suggested that the native tail domain is rich in β‐sheet and β‐turn structures [71], but that the mutated C‐terminal sequence of the tail domain has a substantial increase in α‐helix structure (data not shown). These changes might impair intramolecular or intermolecular interactions of the K85–K35 pair involved in filament formation, thereby causing ectodermal dysplasia of hair and nail type. Interestingly, premixing equal amounts of wild‐type K85 and K85(delCT) constructs followed by cotransfection with the K35 construct restored filament formation. This result might explain the recessive inheritance of the ectodermal dysplasia of hair and nail type caused by the delCT mutation [42]. The K85(delCT) protein might interact with K35 to form an almost wild‐type oligomer structure that incorporates into a growing K85–K35 hair keratin filament, although this hypothesis needs testing.

It has been well documented that head domains of IF proteins are crucial for IF formation *in vitro* and *in vivo* [12, 13, 70]. Certainly, the K85(△Head) mutant that lacks the whole head domain co‐polymerized with K35 but failed to form filaments in transfected SW‐13 cells, suggesting that the head domain of K85 is indispensable for filament formation. However, the R78H mutation in the head domain of K85 did not interfere with filament formation. Arginine residues in the head domains of IF proteins are critical for IF assembly because chemical or enzymatic modification of the guanidino group of arginine residues produces IF proteins that cannot polymerize into IFs *in vitro*, although the exact roles of arginine residues in IF assembly remain obscure [12, 13, 72, 73]. Our results indicate that it is unlikely that the Arg^78^ residue in the head domain of K85 is critically involved in filament formation by the K85–K35 pair. However, the head domains of type II hair keratins have a relatively conserved sequence motif except for the most N‐terminal region [74]. The Arg^78^ residue is located close to the C terminus of the four contiguous nonapeptide quasi‐repeat (Gly‐Gly‐Phe‐Gly‐Tyr‐Arg‐Ser‐Xxx‐Gly)_4_, the physiological significance of which remains unknown. Also, *in vitro* experiments indicate that the head domains of hair keratins act as target sites for certain KAPs: high glycine–tyrosine‐type KAP8.1 binds to the head domain of K85 [68]; high‐sulfur‐type KAP2.1 binds to the head domain of K86 [75]; and high‐sulfur‐type KAP11.1 binds to the head domain of K31, K33 and K34 [76]. It will be of interest to examine whether the R78H mutation affects interactions between the head domain of K85 and KAPs because such interactions play a key part in the growth of proto‐MFs into mature MFs. Further studies are required to understand why the R78H mutation of K85 causes a more severe ectodermal dysplasia of hair and nail type phenotype than that caused by the delCT mutation.

In conclusion, we describe the unique filament development of the human hair keratin pair K85 and K35 in transfected cells. As the first hair keratin pair expressed in a hair‐forming cell, K85 and K35 might have a role in forming an MF nucleus to which other hair keratin oligomers and IFs coalesce. KAPs then infiltrate the nucleus to form mature MFs. Disordered nucleus formation might critically damage MF maturation and cause ectodermal dysplasia of hair and nail type. Further work is required to understand fully the molecular basis of MF formation and to characterize the impacts of hair disease‐related mutations on the MF formation process.

## Conflict of interest

The authors declare no conflict of interest.

## Author contributions

SA conceived and supervised the study. MY, YS and YH prepared the protein expression systems in cultured cells. MY, YS and HN performed fluorescence microscopy. KK and TM provided new tools and performed critical reading of the manuscript. MY, YS and SA wrote the manuscript.

## Data Availability

The data that support the findings of this study are available from the corresponding author upon reasonable request.
